# 

**Published:** 1917-09

**Authors:** 


### TABLE OF CONTENTS

Goitre ---------- 161

Congenital Haemolytic Jaundice in the Adult ---------- 163

The Present Status of Surgical Cleanliness ---------- 167

Wounds of the Lungs in the Present War ---------- 171

The Prevention of Venereal Diseases ---------- 176

Poison Gases in Trench Warfare ---------- 187

The Army's Ten Commandments ---------- 188

Medical Items ---------- 189

